# Autophagy in the Regulation of Placental Development: From Trophoblast Differentiation to Metabolic Stress Adaptation

**DOI:** 10.3390/cells15171552

**Published:** 2026-08-27

**Authors:** Chloe Lee, Hoi Kit Matthew Leung, Yanjie Guo, Sam Chak Sum Wong, Ruiqing Zhang, Lee Tung Chan, Yang Dong, Qingqing Zhang, Ka-Wang Cheung, Philip C. N. Chiu, Cheuk-Lun Lee

**Affiliations:** 1 Department of Obstetrics and Gynaecology, The University of Hong Kong, Hong Kong SAR, China; u3013401@connect.hku.hk (C.L.); lhkm1997@connect.hku.hk (H.K.M.L.); u3010464@connect.hku.hk (Y.G.); sandy666@hku.hk (Y.D.); kelvincheung82@hotmail.com (K.-W.C.); 2 Shenzhen Key Laboratory of Fertility Regulation, The University of Hong Kong-Shenzhen Hospital, Shenzhen 518000, China; zhangqq1@hku.hk; 3 Department of Health Technology and Informatics, The Hong Kong Polytechnic University, Hong Kong SAR, China; 24145987r@connect.polyu.hk (S.C.S.W.); 25027154r@connect.polyu.hk (R.Z.); michellechan.lt@gmail.com (L.T.C.); 4 School of Biomedical Sciences, Li Ka Shing Faculty of Medicine, The University of Hong Kong, Hong Kong SAR, China

**Keywords:** autophagy, placental development, differentiation, syncytialization, invasion, trophoblasts, pregnancy complications, pre-eclampsia, foetal growth restriction, gestational diabetes mellitus, preterm birth, recurrent spontaneous abortion, obstetric antiphospholipid syndrome

## Abstract

Successful pregnancy depends on precise placental development, where trophoblast differentiation, syncytialization, invasion, and adaptation to metabolic stress are critical. Autophagy, a lysosome-mediated degradation pathway, has emerged as an important regulator of cellular homeostasis, yet its integrated role in trophoblast fate and functions has not been comprehensively summarised. This review synthesises current evidence on autophagy’s functions throughout placentation, from trophoblast differentiation to syncytialization and extravillous trophoblast invasion. We examine how autophagy enables cellular remodelling during differentiation, supports metabolic adaptation under hypoxia and nutrient stress, and maintains mitochondrial quality control through selective mitophagy. Autophagy is essential for syncytiotrophoblast formation via endoplasmic reticulum stress-coordinated activation and p53 downregulation. However, its effects on trophoblast invasion are context-dependent, influenced by oxygen tension, autophagic flux completeness, and differentiation state, which can potentially be shaped by parent-offspring genetic conflicts through genomic imprinting. Both excessive and insufficient autophagy contribute to pregnancy complications, including pre-eclampsia, foetal growth restriction, gestational diabetes mellitus, preterm birth, recurrent spontaneous abortion and obstetric antiphospholipid syndrome through distinct molecular mechanisms. Autophagy functions as a dynamically tuned homeostatic mechanism in placental development. Understanding condition-specific autophagy dysregulation is thereby crucial for improving pregnancy outcomes.

## 1. Introduction

A successful pregnancy depends on a regulated series of biological mechanisms, including the implantation of the blastocyst and the subsequent development of the placenta [1]. The placenta is a transient organ responsible for performing diverse functions crucial for intrauterine foetal growth [2]. One of its key functions is facilitating the exchange of nutrients and energy between the mother and the foetus [3]. Moreover, the placenta demonstrates remarkable adaptability to stressful environmental changes within the maternal–foetal interface [4]. Dysregulation or disruptions in these placental mechanisms can significantly impact embryonic growth and development, leading to various pregnancy complications, such as pre-eclampsia (PE), gestational diabetes mellitus (GDM), preterm birth (PTB), and foetal growth restriction (FGR) [5,6].

Central to these critical placental functions lies the intricate process of trophoblast differentiation into distinct cell lineages, each with specialised functions and phenotypes [7]. This differentiation process is accompanied by dynamic molecular changes that determine the identity and function of each trophoblast subtype [8]. Autophagy is a fundamental biological process that maintains cellular homeostasis by degrading cytoplasmic materials and specifically eliminating intracellular pathogens, particularly in stressful situations such as starvation, infection, hypoxia and other forms of physiological stress. Interestingly, autophagy has been shown to be necessary for trophoblast lineage commitment and cellular remodelling during early placentation [8,9,10]. Moreover, impaired autophagy has been consistently linked to placental dysfunction and related pathologies [11,12].

Throughout this review, we differentiate between autophagy induction, referring to increased autophagosome formation, and autophagic flux, referring to completion of the degradation process. Many studies report elevated LC3-II levels as evidence of increased autophagy; however, this may also reflect impaired autophagosome-lysosome fusion. Where possible, we interpret findings based on complementary measures such as p62 abundance and tandem fluorescent reporters.

Previous reviews on placental autophagy documented its involvement in trophoblast functions and placental complications, yet a unifying framework capable of resolving the contradictory findings in related studies is still lacking [13,14,15]. To address these discrepancies, this review introduces a systematic and context-dependent analytical framework that examines the functional consequences of autophagy across different trophoblast subtypes and the autophagic signatures of pregnancy complications across different clinical subtypes and metabolic status, thereby clarifying autophagy’s complex roles across placental development and diseases.

Furthermore, we address the translational limitations of traditional rodent models and organise these insights into a three-tiered translational framework designed to bridge the gap between early biomarker prediction, preclinical mechanistic targeting, and clinical therapeutic interventions to safeguard maternal–foetal health and optimise pregnancy outcomes.

## 2. Early Placental Development

Human placental development begins in the trophectoderm (TE), the outermost layer of the blastocyst. TE cells give rise to a multipotent, self-renewing population known as trophoblast stem cells (TSCs), which serve as the primary progenitor population of all placental lineages [8].

Implantation occurs at approximately 5–6 days post-conception (dpc), allowing stable adherence and infiltration into the decidualized uterine endometrium mediated by carbohydrate-binding glycoproteins such as galectins and selectins, adhesion molecules including integrins, and signalling factors such as heparin-binding EGF-like growth factor (HBEGF) [2,16]. This process triggers bidirectional paracrine signalling, where trophoblasts and decidual cells secrete factors that synchronously regulate trophoblast differentiation and uterine remodelling [17].

Following implantation, TE stem cells give rise to the first trophoblast lineages—early primitive cytotrophoblasts (CTs) and primitive syncytium (PS), with the latter being recognised as the first invasive placental cell type [18,19]. While some CTs remain as a progenitor pool for continuous regeneration, others undergo differentiation to form syncytiotrophoblasts (STs) and extravillous trophoblasts (EVTs) [5,19]. The multinucleated ST layer eventually covers the entire villous surface, serving as the primary biophysical barrier for maternal–foetal exchange and the specialised site for pregnancy-specific hormone production, such as the human chorionic gonadotropin (hCG) [6,10].

Simultaneously, EVTs emerge from the tips of anchoring villi to establish a highly specialised invasive placental cell type essential for establishing the maternal–foetal interface [5,18]. These cells migrate and penetrate deeply into the maternal decidua and myometrium, reaching the maternal spiral arteries. During this process, EVTs acquire endothelial-like properties and participate in the uterine spiral artery remodelling to ensure adequate blood flow to the placenta and support foetal development [9,20].

The success of these differentiation programmes is a crucial prerequisite for a healthy pregnancy. Any disruptions in syncytialization and failures in EVT-mediated vascular remodelling are considered primary drivers in the pathogenesis of early-onset PE and FGR. Thereby, understanding the regulation of these processes is essential for identifying the origins of placental dysfunction.

## 3. Autophagy

Autophagy is a highly conserved lysosome-mediated degradation pathway with the fundamental role of maintaining cellular homeostasis by removing and recycling damaged organelles, misfolded proteins and other cellular components [21]. Autophagy enables cells to adapt to dynamic environmental challenges, including hypoxia, nutrient deprivation, and stress, by reutilising cytoplasmic components to support cell survival and function [22,23]. In brief, autophagy can be differentiated into macroautophagy, microautophagy, and chaperone-mediated autophagy [22]. Among the major types, the most extensively studied macroautophagy (hereafter referred to as autophagy) describes the formation of double-membrane vesicles called autophagosomes, which engulf cellular cargo for lysosomal degradation [21]. While autophagy operates at the basal level continuously in all eukaryotic cells as routine quality control, stress conditions would trigger autophagy as a cytoprotective mechanism [24,25].

Autophagy is molecularly governed by autophagy-related (ATG) proteins that regulate the formation of double-membrane autophagosomes, starting with induction by the unc-51 like autophagy activating kinase-1 (ULK1) kinase complex, followed by nucleation via the class III PI3-kinase complex, membrane elongation and cargo recognition through ubiquitin-like conjugation systems (ATG12–ATG5-ATG16L1 and LC3/ATG8), and ending with fusion of autophagosomes with lysosomes for cargo degradation and recycling [22,26].

At the molecular level, the autophagic pathway is a highly dynamic, multi-stage process known as autophagic flux. This process can be broadly categorised into two major functional phases: upstream initiation and downstream completion, which consist of three sequential steps: autophagosome formation, autophagosome-lysosome fusion and lysosomal degradation. The initiation phase of autophagosome formation involves the generation of the double-membrane autophagosomes, which is induced by unc-51 like autophagy activating kinase-1 (ULK1) kinase complex, followed by nucleation via the class III PI3-kinase complex, membrane elongation and cargo recognition through ubiquitin-like conjugation systems (ATG12–ATG5-ATG16L1 and LC3/ATG8) [22,26]. However, this does not equate to functional cellular clearance. A complete autophagic flux relies on the subsequent fusion of the autophagosomes with lysosomes, where the engulfed cargo and selective autophagic receptors, such as p62, are ultimately degraded and the resulting macromolecules are recycled back into the cytoplasm [21,27]. By understanding this fundamental distinction, it becomes clear that an accumulation of autophagosomes or LC3-II proteins can indicate either a robust initiation of autophagy or a pathological blockade in the downstream phase of fusion and degradation [22].

Upstream of this mechanism, the autophagic process is initiated primarily by the central nutrient and energy sensors, mechanistic target of rapamycin (mTOR) complex and AMP-activated protein kinase (AMPK) [28,29]. Under nutrient-rich conditions, mTOR serves as a negative regulator, inhibiting autophagy [28]. In most cells, AMPK activation under stress promotes autophagy via mTORC1 inhibition and ATG phosphorylation [29]. The tumour suppressor p53 has been shown to negatively regulate autophagy during trophoblast differentiation [30] and its role in modulating autophagy in response to DNA damage has been characterised in other cellular contexts as well [31].

Functionally, autophagy is biologically indispensable in cellular metabolism with diverse roles in development, differentiation and immune regulation [32]. Autophagy is essential to stem cell maintenance and lineage specification by recycling cellular components during differentiation, as well as mitigating oxidative damage and promoting cell survival and tissue integrity under stress [33,34]. Given the physiological significance of autophagy, its dysregulation has been linked to numerous diseases, from metabolic syndromes and neurodegenerative disorders to cancers [35,36,37,38]. An imperative factor in cellular adaptation to metabolic and stress responses is the quality control of mitochondria. Mitophagy is a selective form of autophagy that removes damaged mitochondria, helping to maintain cellular energy balance and decrease oxidative stress by ensuring mitochondria function properly and produce ATP efficiently [39,40].

In placental biology, autophagy plays a pivotal role in supporting the metabolically demanding trophoblasts by facilitating their differentiation, invasion, and adaptation to the dynamic microenvironment at the maternal–foetal interface. The following sections will explore the complex relationship between autophagy and trophoblast biology, highlighting how autophagy supports key processes in normal placental development, including trophoblast differentiation, syncytialization, and invasion. Key studies reporting the significance of autophagy in early placental development are summarised in Table 1. In articles discussing trophoblast invasion, several findings in Table 1 appear contradictory. For instance, Oh et al. reported an increase in trophoblast invasiveness upon autophagy suppression [41], while Tan et al. and Arikawa et al. reported the opposite [42,43]. These discrepancies are not an indication of conflicting results but reflect a multi-layer context-dependency of autophagy in trophoblast invasion, which is systematically discussed in Section 4.3.

In addition, studies of trophoblast autophagy included in this review frequently employ pharmacological modulators including 3-methyladenine (3-MA), chloroquine (CQ), bafilomycin A1 (BAF), and rapamycin. Although they are valuable experimental tools, these agents are not entirely autophagy-specific in function. For instance, 3-MA inhibits both class III and class I PI3K signalling [44]; chloroquine and BAF broadly disrupt lysosomal and endosomal pH and functions beyond autophagosome-lysosome fusion [45,46]; and rapamycin exerts mTOR-dependent effects on protein synthesis and cell growth independent of autophagy induction [47]. Therefore, pharmacological findings should be interpreted together with appropriate autophagic-flux measurements and, where available, concordant genetic approaches targeting core autophagy machinery, such as ATG5, ATG7, ATG16L1, or BECN1. In this review, evidence supported by genetic perturbation and complementary flux assays is considered more mechanistically specific than evidence based on pharmacological modulation alone.

**Table 1:** Key studies studying the role of autophagy in placental development using trophoblast models. Adapted from respective authors as cited in the table. ↑ indicates for promotes and ↓ indicates for depletes. † Pharmacological autophagy modulators (3-MA, CQ, BAF, and rapamycin) are not fully autophagy-specific; see Section 3 for limitations and interpretation alongside genetic and autophagic-flux evidence.

Author	Title	Major Findings	Species/Model System	Key Experimental Contexts	Assay/Autophagy Evidence
Chakraborty et al. [8]	Harnessing Autophagy Network is Essential for Trophoblast Stem Cell Differentiation [PMID: 32143554]	- Inhibition of autophagy ↓ differentiation or cell death	- Mouse (Murine)	- Murine TSCs - Primary: placental tissues from pregnant Swiss albino mice	- LC3-II & p62 Western blots - Lysosomal inhibitors (BAF, CQ) for flux assays † - IF-colocalisation (LC3/p62)
Tozawa et al. [10]	Upregulation of Autophagy During the Differentiation of Primary Human Term Cytotrophoblast Cells into Syncytial Cells: Ultrastructure Analysis [PMID: 39941088]	- Autophagy upregulates when CTs progressively differentiate into STs to support cell fusion and syncytial formation - Perinuclear accumulation of extremely large autophagosomes/autolysosomes and organelle-specific autophagy are observed in STs	- Human	- Primary: CTs isolated from human term placentas	- Transmission electron microscopy (TEM) for autophagosome/autolysosome ultrastructure
Bastida-Ruiz et al. [48]	The fine-tuning of endoplasmic reticulum stress response and autophagy activation during trophoblast syncytialization [PMID: 31501418]	- Co-activation of unfolded protein response (UPR) and autophagy ↑ syncytialization - Disrupted UPR ↓ autophagy activation and subsequent trophoblastic fusion, differentiation and cell survival	- Human	- Cell line(s): BeWo - Primary: villous CTs (vCTs) isolated from human placentas	- LC3-II & BECN-1 Western blots - Aciridine Orange staining (acidic vacuole assay) - Lysosomal inhibitors (BAF, CQ) † - Autophagic activators (Trichostatin A (TRI)/Valproic acid (VA)) - Geneitic manipulation (BECN-1 plasmid & siRNA)
Gauster et al. [30]	Downregulation of p53 drives autophagy during human trophoblast differentiation [PMID: 29080089]	- BeWo cells were induced for p53 downregulation, triggering autophagy activation during ST differentiation - p53 downregulation-driven autophagy facilitates cellular remodelling and organelle reorganisation	- Human	- Cell line(s): BeWo, JAR - Primary: human placenta tissues and explants	- LC3-II & p62 Western blots - Tandem LC3 reporters (GFP-LC3)/Flux assays (with leupeptin) † - IF microscopy for LC3B puncta quantification
Arikawa et al. [43]	Galectin-4 expression is downregulated in response to autophagy during differentiation of rat trophoblast cells [PMID: 27572741]	- Autophagy ↓ Galectin-4 (Gal-4) during Rcho-1 differentiation - Gal-4 downregulation is essential for trophoblast invasion by altering epithelial characteristics, dissociating cell–cell interactions and gaining an invasion phenotype	- Rat	- Cell line(s): Rcho-1 - Primary: placenta tissues from pregnant Wistar Hannover rats	- LC3-II & p62 Western blots - Lysosomal inhibitors (BAF) for flux assays † - Autophagy inhibitors (3-MA)
Oh et al. [41]	Autophagy regulates trophoblast invasion by targeting NF-κB activity [PMID: 32820194]	- ↓ Autophagy → ↑ NF-κB activity + p65 expression → ↑ Akt + MMP2/9, ↓ sFlt-1 + TNF-α → ↑ invasion	- Human	- Cell line(s): JEG-3, HTR-8/SVneo	- LC3-II, p62, BECN-1 & ATG5 Western blots - Lysosomal inhibitors (BAF) † - Autophagy acitvators (Rapamycin) † - Genetic manipulation (shRNA ATG5/BECN-1)
Chu et al. [49]	Amnion-Derived Mesenchymal Stem Cell Exosomes-Mediated Autophagy Promotes the Survival of Trophoblasts Under Hypoxia Through mTOR Pathway by the Downregulation of EZH2 [PMID: 33304896]	- AD-MSC exosomes treatment ↑ trophoblast proliferation and autophagy under hypoxic conditions - AD-MSC exosomes treatment ↓ EZH2 and mTOR signalling → ↑ autophagy and trophoblast survival	- Human	- Cell line(s): JEG-3, HTR-8 - Primary: primary amnion-derived mesenchymal stem cells (AD-MSCs) - Hypoxia: 37 °C, 2%O_2_/5%CO_2_/93%N_2_ (Invivo2 Hypoxia Workstation)	- LC3-II, p62 & BECN-1 Western blots - Lysosomal inhibitors (BAF) under hypoxia for flux assays †
Chu et al. [50]	Chorionic villus-derived mesenchymal stem cell-mediated autophagy promotes the proliferation and invasiveness of trophoblasts under hypoxia by activating the JAK/STAT3 signalling pathway [PMID: 34645519]	- CV-MSC treatment ↑ trophoblast proliferation, invasion and autophagy under hypoxic conditions - JAK2/STAT3 activation is the prerequisite for CV-MSCs-mediated autophagy to support trophoblast functions	- Human	- Cell line(s): JAR, JEG-3, HTR-8 - Primary: human placental explants - Hypoxia: 37 °C, 2%O_2_/5%CO_2_/93%N_2_ (Invivo2 Hypoxia Workstation)	- LC3-II, p62 & BECN-1 Western blots - Lysosomal inhibitors (BAF) & autophagy inhibitors (3-MA) under hypoxia †
Nakashima et al. [12]	Endoplasmic reticulum stress disrupts lysosomal homeostasis and induces blockage of autophagy flux in human trophoblasts [PMID: 31391477]	- ER stress impairs both lysosomal function and autophagic flux ER-stress-induced lysosomal dysfunction is linked to reduced serum LAMP1/β-galactosidase (β-gal) in PE and FGR	- Human	- Cell line(s): BeWo, HchEpc1b, TCL1 - Primary: primary human trophoblasts (PHTs) - Serum samples from non-pregnant, normally pregnant and patients with PE or FGR	- LC3-II & p62 Western blots - Lysosomal inhibitors (BAF, CQ) for flux assays †
Capatina et al. [51]	Excessive endoplasmic reticulum stress drives aberrant mouse trophoblast differentiation and placental development, leading to pregnancy loss [PMID: 34269420]	- Chronic ER stress ↑ UPR signalling pathways, leading to premature differentiation, FGR and pregnancy loss - Tauroursodeoxycholic acid (TUDCA) rescues trophoblast population and decreases early pregnancy loss in chronic ER stress mice (*Eif2s1*^+/tm1RjK^)	- Mouse (Murine)	- Mouse TSCs - Animal: C57BL/6 J mice, Eif2s1+/tm1RjK mutant mice - Primary: blastocysts and placental tissues from Eif2s1+/tm1RjK mice	- LC3-II & p62 Western blots - IF microsopy for LC3B puncta quantification - Genetic manipulation for *Atg* gene expression analysis
Tan et al. [42]	Autophagy suppression of trophoblast cells induces pregnancy loss by activating decidual NK cytotoxicity and inhibiting trophoblast invasion [PMID: 32398034]	- ↓ Autophagy in trophoblasts → ↑ IGF-2 secretion, ↓ PEG10 expression in RSA patients - ↑ IGF-2 secretion → ↑ decidual NK (dNK) cell differentiation and cytotoxicity in trophoblasts - PEG10 reduction ↓ trophoblast invasion	- Human - Mouse	- Cell line(s): HTR-8/SVneo - Primary: villi tissues from RSA - Animal: C57BL/6 J mice	- LC3-II & p62 Western blots - Lysosomal inhibitors (BAF) † - Autophagy inhibitors (3-MA) † - Genetic manipulation (shRNA ATG5/LC3B)
Ji et al. [52]	MiR-193b inhibits autophagy and apoptosis by targeting IGFBP5 in high-glucose induced trophoblasts [PMID: 33010605]	- ↓ miR-193b expression in GDM patients - HG → ↓ miR-193b → ↑ IGFBP5 → ↑ autophagy → ↑ apoptosis & ↓ proliferation/cell growth - Restoring miR193b or silencing IGFBP5 (siGFBP5) reverses the HG-induced autophagy, apoptosis and cytotoxic effects, supporting trophoblast survival	- Human	- Cell line(s): HTR-8/SVneo - Primary: peripheral blood and placental tissues from GDM patients and NP	- LC3-II & p62 Western blots - Autophagy activators (Rapamycin) & inhibitors (3-MA) † - Genetic manipulation (siIGFBP5) under HG
Ji et al. [53]	High glucose-induced p66Shc mitochondrial translocation regulates autophagy initiation and autophagosome formation in syncytiotrophoblasts and extravillous trophoblast [PMID: 38643181]	- HG induces mitochondrial p66Shc translocation, initiating autophagy via mito-associated ER membrane (MAMs) and PINK1-PRKN-mediated mitophagy - p66Shc activates cGAS-STING pathway, mediating autophagosome formation and inducing autophagy and cellular senescence - ↓ p66Shc → ↓ HG-induced autophagy and cellular senescence, potentially modulating GDM-related placental dysfunctions	- Human	- Cell line(s): BeWo, HTR-8/SVneo - Primary: placental tissues from GDM patients and NP	- LC3-II & p62 Western blots - Mitophagy flux markers (e.g., PINK1, PRKN) - TEM for autophagosome and mitochondrial ultrastructure - Genetic manipulation (si-ATG5) under HG
Wang et al. [54]	Silencing DAPK3 blocks the autophagosome-lysosome fusion by mediating SNAP29 in trophoblast cells under high glucose treatment [PMID: 31811899]	- ↑ DAPK3 expression in GDM placentas and HG-treated trophoblasts - ↓ DAPK3 → ↓ autophagosome-lysosome fusion under HG conditions - ↓ DAPK3 → ↑ EVT invasion	- Human	- Cell line(s): HTR-8/SVneo - Primary: placental tissues from GDM patients and NP	- LC3-II & p62 Western blots - Tandem LC3 reporters (mRFP-GFP-LC3)/Flux assays - SNARE assembly assay (co-immunoprecipitation (co-IP) of STX17-SNAP29-VAMP8) - Genetic manipulation (p66Shc knockdown (p66Shc-KD) & siDAPK3) under HG
Cao et al. [55]	ATG16L1 governs placental infection risk and preterm birth in mice and women [PMID: 28018968]	- ATG16L1-dependent autophagy protects STs from bacterial infection - ATG16L1 deficiency or dysfunction is linked to increased placental infection and inflammation-induced PTB - Rapamycin (inducer of autophagy) ↓ bacterial loads in human placental explants	- Human - Mouse	- Cell line(s): BeWo - Primary: PHTs isolated from human term placentas, human placental tissues, mouse placental explants - Animal: Atg16L1 hypomorphic mice	- LC3-II & p62 Western blots - IF microsopy for LC3B puncta quantification - Lysosomal inhibitors (BAF) for flux assays † - Autophagic activators (Rapamycin) †
Yang et al. [29]	Modulation of Autophagy Through Regulation of 5′-AMP-Activated Protein Kinase Affects Mitophagy and Mitochondrial Function in Primary Human Trophoblasts [PMID: 33619701]	- AMPK regulation modulates autophagy, mitophagy and mitochondrial fusion/fission in PHTs - Both AMPK activation and inhibition ↓ autophagosome-lysosome fusion and impairs mitophagy, leading to disrupted mitochondrial quality control, altered network homeostasis; ↑ mitochondrial biogenesis	- Human	- Primary (ex vivo): primary trophoblasts isolated from human term placentas	- LC3-II & p62 Western blots - Mitophagy flux assays (colocalization of LC3 & TOM20) - Lysosomal inhibitors (CQ) for flux assays †
Viola et al. [28]	mTOR is an essential gate in adapting the functional response of ovine trophoblast cells under stress-inducing environments [PMID: 39341011]	- mTOR inhibition (Rapamycin)-induced autophagy acts as a survival mechanism due to full mTOR inhibition - During nutrient deprivation, mTOR is initially inhibited to promote autophagy, but nutrient reintroduction reactivates it to balance autophagy	- Sheep (Ovine)	- Cell line(s): oTr - Primary: primary ovine trophoblast cells (oTCs)	- LC3-II Western blots - Autophagy activators (Rapamycin) †
Carvajal et al. [9]	The autophagy process and oxidised LDL independently modulate the invasion and differentiation of extravillous trophoblastic cells to an endothelial-like phenotype in normoxia [PMID: 39527856]	- Autophagy supports endothelial differentiation but not invasion - ox-LDLs do not impact autophagy but both ox-LDLs and autophagy influence placentation in early pregnancy and PE	- Human	- Cell line(s): HTR-8/SVneo - Primary: human placenta tissues from PE patients & NP	- LC3-II & p62 Western blots - Lysosomal inhibitors (BAF) for flux assays † - IF microscopy for LC3B puncta quantification

## 4. Autophagy in Trophoblast Differentiation, Syncytialization and Invasion

### 4.1. Autophagy as a Driver of Trophoblast Differentiation

Trophoblast differentiation involves a series of dynamic transformations crucial for the development of specialised trophoblast subtypes, comprising alterations in morphology, transcription factors, and overall cellular properties [8]. Emerging evidence confirms that autophagy, a key catabolic process, is significantly integrated into trophoblast differentiation, particularly in the fusion and differentiation of CTs into STs, known as syncytialization. For instance, during the transition from CTs or TSCs, there is a stable increase in ULK1 initiation complex and autophagosome maturation markers such as ATG5, ATG7 and LC3-II [8,10].

Crucially, the inhibition of autophagy before the induction of differentiation severely impairs lineage commitment. In murine models, suppressing autophagy results in a significant reduction in the expression of specific differentiation markers, such as *Prl3d1* and *Tpbpa*, and a reduction in the nuclear size of differentiated cells [8]. These findings indicate that autophagy is developmentally intertwined with trophoblast differentiation, providing the metabolic and structural remodelling necessary for cells to adopt their differentiated fate. In human placentation, detailed ultrastructural evidence supports this “remodelling” role, as CTs progressively differentiate into STs, revealing a unique perinuclear accumulation of extremely large autophagosomes/autolysosomes. These ‘gigantic’ compartments are physically necessary, reflecting the extensive degradation of pre-existing mononucleated structures required to facilitate the massive organelle turnover and cytosolic rearrangements to form a terminal differentiated state [10]. Collectively, these studies highlight that properly functioning autophagy is an indispensable prelude to trophoblast lineage commitment, providing the essential protein turnover and metabolites required for successful differentiation. However, while the instructive role of interrogated autophagy in EVT differentiation remains unexplored, basal autophagy is indispensable for EVTs to acquire a differentiated phenotype for proper maternal vascular remodelling [9].

### 4.2. Triggers and Gating of Syncytialization: The UPR-p53-Autophagy Axis

The transition of mononucleated CTs into a multinucleated ST is a major structural process that requires the activation of internal stress-response pathways to facilitate cellular remodelling. In the healthy placenta, the activation of the UPR and the downregulation of p53 serve as the essential coordinated signals that harness the autophagic machinery as the primary driver for this differentiation process [30,48].

In a healthy pregnancy, moderate activation of the UPR serves as a physiological signal to trigger autophagy required for fusion and differentiation. Experimental evidence shows that inducing the UPR is sufficient to increase the fusion index and β-hCG secretion, while inhibiting UPR pathways effectively blocks syncytialization by preventing the upregulation of autophagy markers [48]. Parallel to this, the downregulation of p53 acts as a crucial molecular gate, with its decrease being an essential requirement specific to the functional remodelling of the STs. Gauster et al. demonstrated that while cells with stabilised p53 may still express differentiation markers like syncytin-1, they fail to undergo the structural transition that only active autophagic machinery can facilitate, leading to inhibited syncytialization and excessive apoptosis [30].

To conclude, these studies demonstrate that the coordinated regulation of the UPR-p53 axis is necessary to activate the robust autophagic flux required for functional syncytialization, proving that this dynamic cellular change cannot be completed without the cellular revitalisation provided by the autophagy machinery.

### 4.3. Context-Dependent Roles of Autophagy in Trophoblast Invasion

The impact of autophagy on trophoblast invasion has often appeared contradictory across studies. These discrepancies in autophagy’s role in invasion can be reconciled by recognising that it is governed by several sequentially nested layers of context. Firstly, the broad environmental frame is oxygen tension. Secondly, the completeness of autophagic flux determines whether the environmental signal is fully executed. Thirdly, the functional outcome is further specified by the particular molecular targets being degraded and the differentiation state of trophoblasts. Together, these three layers explain why autophagy does not exert a binary effect on invasion but rather regulates placentation in a context-dependent manner.

#### 4.3.1. Context of Oxygen Tension: Physiological Hypoxia vs. Normoxia

The oxygen concentration at the maternal–foetal interface appears to associate with divergent autophagic outcomes in trophoblast invasion, although its interpretation is complicated by differences in experimental approaches.

Under physiological hypoxic conditions at 2% oxygen (O_2_), which mimics an early placentation environment, autophagy serves as a crucial pro-invasive mechanism. In this context, exposure to conditioned medium (CM) from chorionic villus-derived mesenchymal stem cells (CV-MSCs) significantly enhances the invasive capability of trophoblasts from cell lines JAR, JEG-3, and HTR-8 in Transwell assays by promoting autophagy via the JAK2/STAT3 pathway [50]. Similarly, exosomes derived from amnion-derived mesenchymal stem cells (AD-MSCs) promote trophoblast survival and proliferation of HTR-8/SVneo cells under hypoxic stress by downregulating enhancer of zeste homology 2 (EZH2); this downregulation inactivates mTOR and subsequently enhances autophagic activity [49].

Meanwhile, under standard culture normoxic conditions (at 20% O_2_), basal autophagy appears to function as a regulatory ‘brake’ to prevent pathological excessive invasion. For instance, Oh et al. showed that blocking autophagy initiation by silencing ATG5 markedly enhances trophoblast invasiveness by 54% under normoxic conditions [41]. Mechanistically, autophagy under normoxia continuously degrades pro-inflammatory components of the nuclear factor-kappa B (NF-κB) signalling pathway, including its p65 subunit. When autophagy is inhibited, the resulting accumulation of p65 hyperactivates NF-κB, which subsequently upregulates pro-invasive matrix metalloproteinases (MMP) such as MMP-2/9 [41].

However, it should be borne in mind that direct comparison of the above studies is complicated by their methodological differences. The hypoxia studies enhanced autophagy through exogenous MSC-derived conditioned medium and exosomes, whereas the normoxia study suppressed autophagy via genetic ATG5 knockdown [41,49,50]. The studies above illustrate that oxygen tension is associated with divergent autophagic roles in trophoblast invasion, and yet, interpretation should be necessarily cautious given the heterogeneity of experimental approaches across studies. Despite these limitations, the studies nevertheless point towards the notion that a properly functioning autophagy process is necessary for, or enhances, this process by supplying energy and cellular components, especially under the physiologically hypoxic conditions of early pregnancy, while simultaneously serving as a molecular regulator to prevent over-invasion under normoxia.

Rather than viewing the distinct invasive responses of trophoblasts under hypoxia and normoxia as mere experimental discrepancies, we propose that this context-dependency reflects an evolved, functional navigation mechanism that reconciles the apparently contradictory findings. During early placentation, EVTs must migrate away from the anchoring villi, traverse the decidual stroma and actively locate the maternal spiral arteries to initiate vascular remodelling [5,6]. As these cells travel through the hypoxic decidual microenvironment, the stabilised hypoxia-inducible factor 1-alpha (HIF-1α) upregulates autophagy to meet the metabolic demands and cellular remodelling required to sustain invasion [41,56]. We hypothesise that when invading EVTs successfully breach a maternal arteriole and encounter highly oxygenated maternal blood, this sudden oxygen shift downregulates autophagic activity, acting as an environmental “stop signal” that halts further migration and instead promotes a “stop-and-remodel” phenotype, in which cells settle in situ, undergo endovascular differentiation, and initiate arterial plugging and remodelling rather than continued invasion [57]. Under this framework, autophagy functions as a proposed oxygen-gated switch that reframes the invasion-related discrepancies in Table 1 as complementary snapshots of a single navigational mechanism, a hypothesis warranting direct testing via oxygen-gradient invasion assays with autophagy flux reporters.

#### 4.3.2. Context of Autophagy Flux Completeness: Initiation vs. Fusion

Building on the framework of oxygen tension established above, a second layer of context concerns not whether autophagy is active, but how completely the process is executed. A significant source of debate regarding trophoblast invasiveness centres on the specific stage of the autophagic process being disrupted, namely, the initiation of autophagosome formation versus its fusion with lysosomes.

Inconsistent findings in the effects of autophagy initiation blockade highlight the ‘double-edged’ nature of autophagy in the placenta. Whereas Oh et al. found that blocking autophagy initiation via short hairpin RNA (shRNA) targeting ATG5 or beclin-1 (BECN-1) increases invasion in trophoblast cell lines, they noted that their experiments were conducted under naïve conditions in normoxia [41]. From this, it is acknowledged that this discrepancy likely originates from the specific experimental context, confirming that autophagy does not have a binary effect but rather functions as a metabolic rheostat to modulate the depth of placentation.

Supporting this contextual view, other studies show that the invasion process fails without the autophagy ‘metabolic driver’ when physiological stress or specific molecular switches are involved. For instance, in both human HTR-8/SVneo cells and rat Rcho-1 cells, inhibiting the initiation stage with 3-methyladenine (3-MA) or silencing ATG5 (siATG5) leads to a significant reduction in invasiveness [42,43]. These anti-invasive effects are primarily due to abnormalities in key molecular switches, such as the downregulation of paternally expressed gene 10 (PEG10) during autophagy inhibition. This highlights that the initiation of autophagy is a prerequisite for trophoblast invasion under the physiological stresses encountered during early pregnancy.

Interestingly, investigating the late stage of autophagy, which is the fusion of autophagosomes with lysosomes, reveals that autophagy regulates vascular remodelling independently of invasiveness. Treatment with Bafilomycin A1 (BAF) to block autolysosome formation does not significantly impact the initial penetration capacity of HTR-8/SVneo cells; however, it severely impairs their differentiation into an endothelial-like phenotype, leading to a reduction of up to 60% in reticular network formation [9]. This distinct phenomenon suggests that while autophagy initiation may promote trophoblast invasion, the final-stage fusion is specifically required for the structural transition needed to remodel the maternal spiral arteries [9,58].

Taken together, these studies reveal that the completeness of autophagic flux, from initiation to lysosomal degradation, is a crucial requirement for not only maintaining the function of the invasive phenotype but also the proper vascular remodelling capacity of trophoblasts. Beyond the stage of autophagic flux and environmental oxygen tension, the specific molecular targets through which autophagy exerts its effects on trophoblast invasion represent a third layer of context dependency.

#### 4.3.3. Context of Specific Molecular Targets and Differentiation States

Beyond oxygen environment and flux completeness, the third layer of context concerns the specific molecular targets that autophagic degradation acts upon, which further shapes the invasion outcome depending on the differentiation state of the trophoblast. Autophagy regulates trophoblast invasion by modulating the expression of key molecular switches that govern the transition of trophoblasts from an epithelial to an invasive phenotype.

In Arikawa et al.’s study, they found that Gal-4 is highly expressed in the proliferative state of rat Rcho-1 cells but is significantly downregulated during the transition to an invasive phenotype [43]. To demonstrate that autophagy acts as the upstream regulator of this process, research shows that inhibiting autophagy with 3-MA or BAF completely abolishes the downregulation of Gal-4, while overexpressing Gal-4 directly interferes with cell enlargement and mobility required for invasion. This suggests that autophagy-mediated degradation of Gal-4 allows trophoblasts to lose their epithelial character and acquire the mobility necessary for successful placentation.

Clinically, a reduced autophagic flux at the maternal–foetal interface is a known hallmark of RSA [42]. Research using HTR-8/SVneo cells shows that inhibiting autophagy with 3-MA or siATG5 leads to a significant reduction in PEG10 levels, a known promoter of trophoblast mobility, and an increase in the secretion of insulin-like growth factor 2 (IGF-2), enhancing decidual natural killer (dNK) cell cytotoxicity and impairing trophoblast invasion [42]. From an evolutionary genetics perspective, this pathological cascade is best understood through the lens of David Haig’s Kinship Theory of genomic imprinting, which describes the parent-offspring genetic conflict at the maternal–foetal interface [59,60]. Both PEG10 and IGF-2 are classic paternally expressed imprinted genes that have evolved to promote trophoblast invasion and maternal resource extraction to support foetal development. In contrast, maternal evolutionary interests aim to limit this resource drain to ensure maternal survival and preserve energy for future pregnancies [60]. Under normal physiological conditions, autophagy acts as a central metabolic mediator that manages this conflict. When autophagic flux is compromised, the downregulation of PEG10 and the abnormal oversecretion of IGF-2 run unchecked, triggering decidual NK cells to act as a destructive maternal immunological brake that ultimately rejects the developing embryo [42]. Thus, a properly functional autophagic network is essential to keep the paternal drive within homeostatic limits that do not trigger maternal immune rejection that would otherwise lead to pregnancy loss.

Interestingly, emerging evidence identifies an independent stressor that impairs placentation without involving the autophagic network. In HTR-8/SVneo cells, exposure to oxidised low-density lipoproteins (ox-LDLs), a risk factor for PE, significantly inhibits cell invasiveness and reticular network formation [9]. However, unlike other metabolic stressors, ox-LDL treatment does not affect the levels of autophagic markers such as LC3-II and p62, and its inhibitory effects are neither additive nor synergistic when combined with autophagy inhibition. This implies that while autophagy is an indispensable driver of invasion and vascular remodelling, other maternal factors, like lipid oxidation, can disrupt placentation via non-autophagic mechanisms.

**Figure 1:**
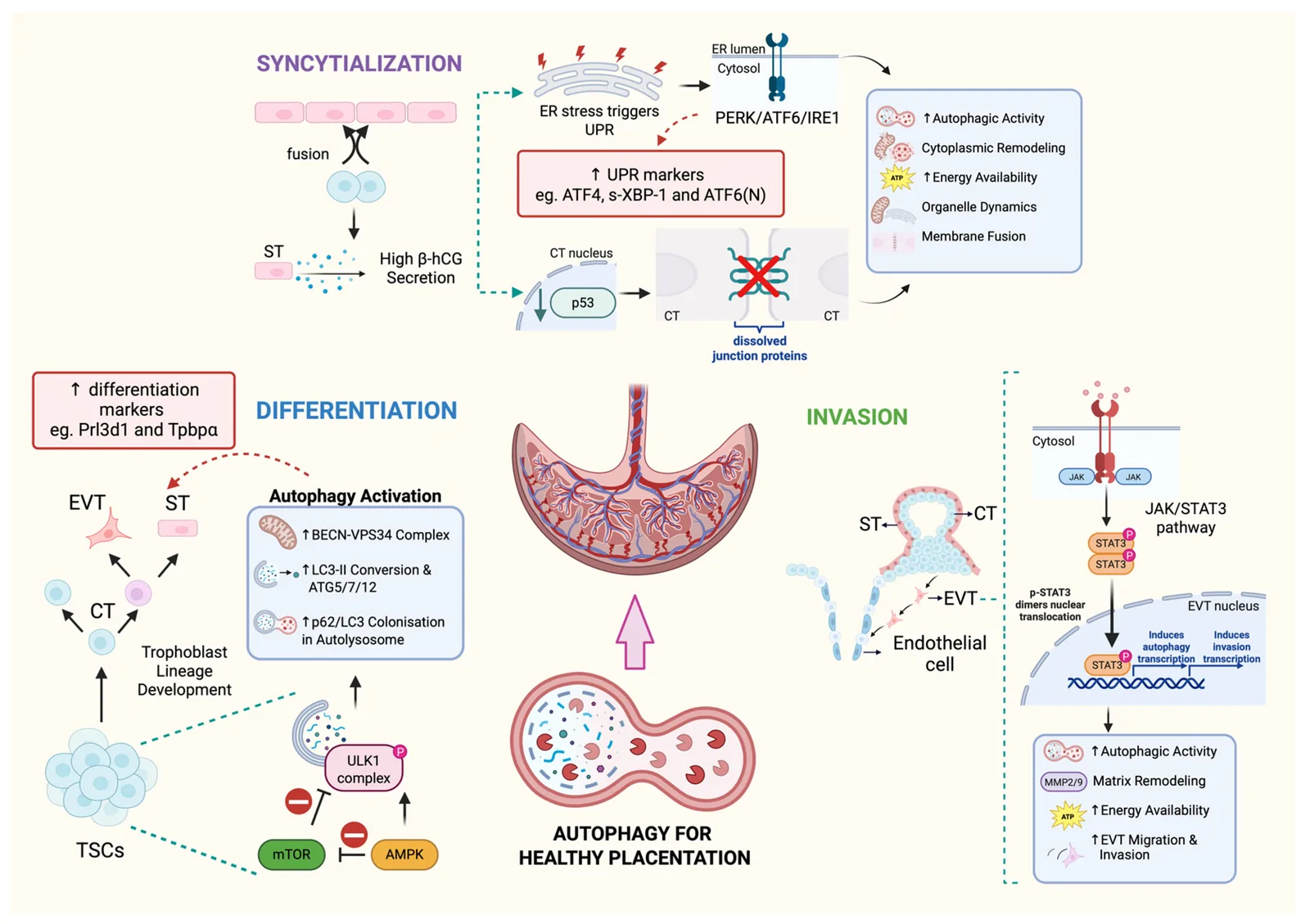
Summary of the role of autophagy initiation and autophagic flux in healthy placentation by supporting trophoblast differentiation, syncytialization and invasion.

In summary, these studies further support that autophagy is a central regulator of proper placental development, fine-tuning the expression of key developmental proteins to ensure proper trophoblast functions. Figure 1 summarises the three key trophoblast pathways, differentiation, syncytialization and invasion, and indicates the molecular pathways through which autophagy supports each.

## 5. Autophagy in Stress, Metabolic Regulation and Mitochondrial Function

Autophagy enables trophoblast adaptation to placental stressors, including ER stress, oxidative stress, hypoxia, and high glucose exposure, by maintaining cellular homeostasis through recycling of damaged components. Its tight regulation preserves metabolic balance, while dysregulation contributes to pathological placental outcomes. Moreover, these autophagy-dependent adaptations operate within an integrated stress-response network in which ER stress, hypoxia, hyperglycaemia and mitochondrial dysfunction converge on common signalling nodes, such as mTORC1, AMPK and the UPR, such that perturbation in one compartment can sensitise trophoblasts for pathological responses in another.

### 5.1. Endoplasmic Reticulum (ER) Stress

In particular, ER stress is a notable autophagy inducer in trophoblasts. ER stress builds up physiologically from the high protein synthesis demand of trophoblast differentiation, syncytialization and placental growth [12,48,51]. Yet, when the folding capacity of the ER is overwhelmed, misfolded proteins accumulate and activate the UPR with three canonical branches—PERK, ATF6, and IRE1—to collectively restore ER homeostasis or trigger apoptosis if the stress is unresolved. Physiologically, moderate ER stress activates autophagy in a fine-tuned coordination to clear damaged organelles and misfolded proteins, which is crucial to the formation of STs [48]. Meanwhile, excessive and unresolved ER stress disrupts lysosomal homeostasis, leading to the blockade of autophagic flux, which inhibits the lysosome-dependent degradation processes central to autophagy [12]. The excessive ER stress and autophagy inhibition cooperatively impair trophoblast viability and disrupt syncytialization. Consistently, in the chronic ER stress model (*Eif2s1*^tm1RjK^), elevated ER stress results in a significant reduction in the Gcm1+ and Syna+ ST populations required for proper labyrinth formation, ultimately causing mid-gestational pregnancy loss [51]. The fine-tuning of ER stress responses, particularly downstream of the UPR, and the sequential autophagy activation are thereby essential for trophoblast viability and placental integrity, whereas unchecked ER stress could exacerbate pre-gestational metabolic or gynaecological conditions through impaired autophagic clearance.

### 5.2. Hypoxia and Nutrient Deprivation

Besides being a driver of trophoblast invasion as discussed in the previous section, hypoxia also acts as a metabolic stressor that engages autophagy for trophoblast survival and functional adaptation. Hypoxia prevails in the developing placenta, where trophoblast survival is supported by autophagy activated through mTOR inhibition and JAK2/STAT3 signalling [49,50]. The autophagic metabolic reprogramming is vital for withstanding low-oxygen and nutrient-insufficient environments during early pregnancy. However, severe hypoxia is found to be associated with PE and adverse pregnancy outcomes. AD-MSC exosomes were reported to activate autophagy and promote trophoblast survival under hypoxic conditions via EZH2 and mTOR signalling inhibition [49]. In addition, exposure to human CV-MSC supernatant enhanced trophoblast proliferation and invasion by upregulating autophagy through JAK2/STAT3 signalling [50]. Similarly, an ovine trophoblast study revealed that mTOR also regulates autophagy under a low-nutrient environment to achieve nutrient recycling [28]. This is where mTOR is initially inhibited to increase autophagosome synthesis; however, prolonged starvation and subsequent nutrient recycling reactivate mTOR to prevent excessive degradation and support trophoblast functionality. Collectively, these findings highlight the modulatory role of autophagy in supporting trophoblast functions against the challenges driven by fluctuations in utero conditions.

### 5.3. High Glucose (HG) Exposure

In contrast to nutrient deprivation, metabolic stress, exemplified by HG exposure that mimics diabetic conditions, exaggerates autophagy and dysregulates trophoblast functions. Oxidative stress and mitochondrial dysfunction enhanced by HG levels are shown to increase LC3-II/LC3-I ratios and decrease p62 in EVT-like HTR8 cells [52,61]. This markedly enhanced, excessive autophagic flux ultimately overconsumes organelles and critical cellular proteins, which triggers caspase-dependent apoptosis and leads to reduced proliferation and invasive capacity of EVTs. A recent study revealed that downregulation of miR-193b relieves repression of its target, insulin-like growth factor binding protein 5 (IGFBP5), promoting mTORC1-linked autophagy and enhancing caspase-3 and caspase-9 activated apoptosis in HG-treated HTR8 cells, thereby exacerbating trophoblast death and shallow placental implantation under hyperglycaemic stress from the potential crosstalk between autophagy and apoptosis [52,62]. In addition, HG stimulates the mitochondrial translocation of the redox adaptor p66Shc protein, which increases mitochondrial ROS, lowers mitochondrial membrane potential, and promotes MAM formation for the initiation of autophagy and autophagosome formation in STs and EVTs [53]. These findings support a direct linkage between hyperglycaemia-driven metabolic stress and mitochondrial dysfunction, as p66Shc-dependent mtDNA release and cGAS-STING activation sustain dysregulated autophagy and mitophagy, contributing to trophoblast dysfunction under HG exposure [53]. Furthermore, death-associated protein kinase-3 (DAPK3) facilitates autophagosome-lysosome fusion in EVTs under HG conditions, whereas silencing DAPK3 (siDAPK3) blocks this fusion and effectively rescues the aberrant EVT invasion caused by HG [54]. Across different models, the above studies demonstrated that HG-driven autophagy is maladaptive and is directly linked to trophoblast apoptosis and impaired invasion.

### 5.4. Mitophagy as a Regulator of Trophoblast Mitochondrial Functions

Following the previous discussion, mitochondrial integrity is paramount for trophoblast energy metabolism and adaptation to stress responses. The specialised form of autophagy, mitophagy, safeguards a healthy mitochondrial network by selectively removing dysfunctional or redundant mitochondria, thus preventing the excessive build-up of reactive oxygen species (ROS) and oxidative damage. In PHTs, perturbing AMPK signalling disrupts autophagic flux and impairs mitophagy, leading to defective mitochondrial quality control [29]. Apart from the modulation of autophagosome-lysosome fusion and eventually mitophagy, AMPK signalling also targets peroxisome proliferator-activated receptor γ coactivator-1 alpha (PGC-1α) and peroxisome proliferator-activated receptor γ (PPARγ) to compensatorily stimulate mitochondrial biogenesis and mitochondrial fatty acid oxidation [29]. Moreover, HG induces the mitochondrial translocation of the redox adaptor p66Shc, which drives excessive mitophagy through the PTEN-induced kinase 1 (PINK1) and Parkin (PRKN) pathway [53]. In particular, PINK1 and PRKN, which target and tag damaged mitochondria for autophagic degradation, are significantly upregulated in the placenta of GDM patients [53]. The maintenance of a healthy mitochondrial network via mitophagy is thereby essential for providing the energy supply and redox balance required for trophoblast viability. Disruptions to this tightly orchestrated autophagic mechanism can compromise trophoblast function and contribute to pregnancy complications.

### 5.5. Integrated Stress Responses: A Network View

**Figure 2:**
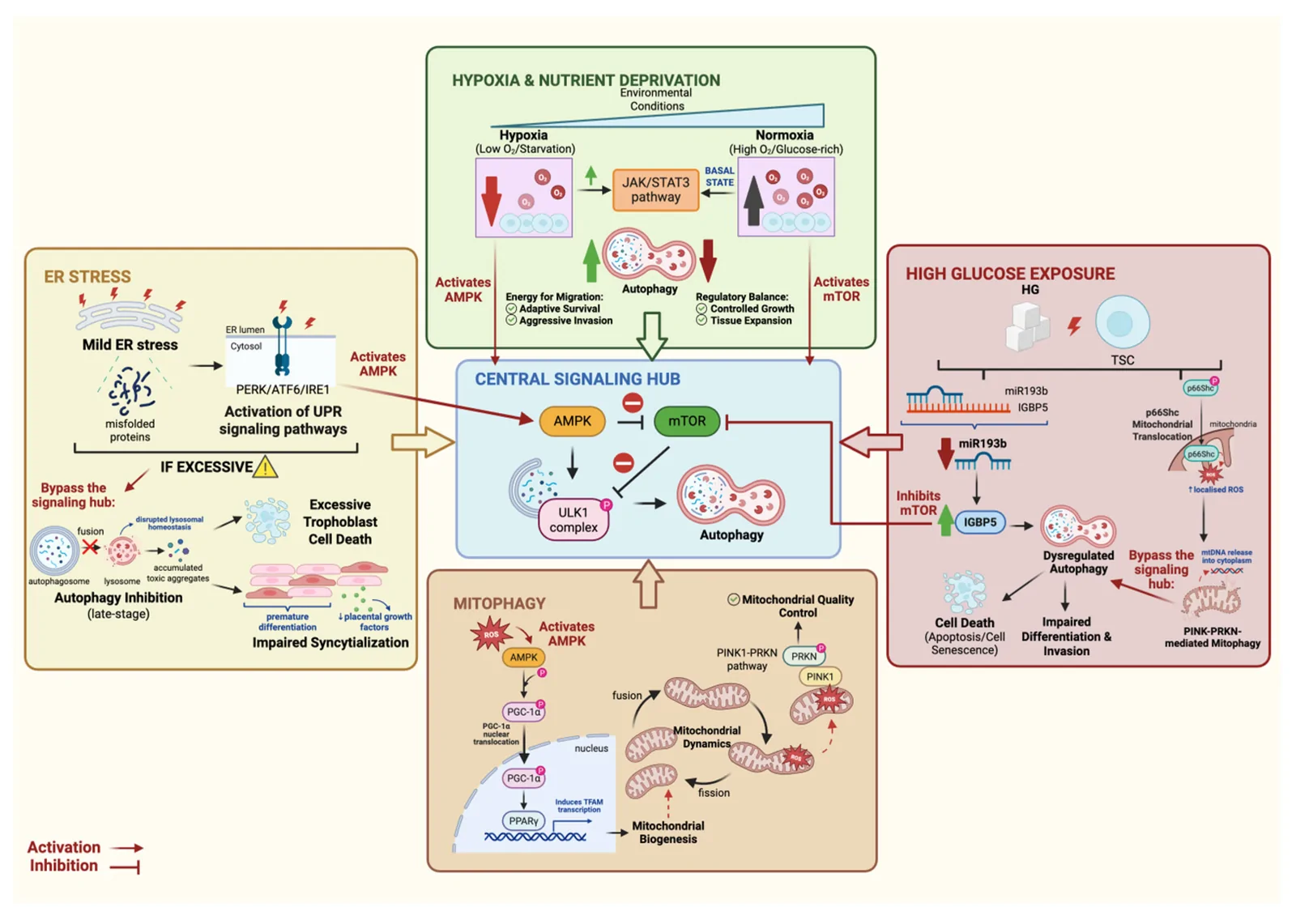
Summary of the role of autophagy in adaptation to metabolic stress and mitochondrial functions of trophoblasts from key studies.

While discussed separately above, ER stress, hypoxia, hyperglycaemia, and mitochondrial dysfunction are not isolated events but interconnected through shared signalling nodes, particularly mTORC1, AMPK, and the UPR. For instance, HG-induced mitochondrial ROS can exacerbate ER stress, while ER stress-induced autophagy blockade further impairs mitochondrial quality control [53]. This network perspective is essential for understanding how mild stress in one compartment can sensitise trophoblasts to pathology in another. The role of autophagy in metabolic stress adaptation and mitochondrial functions is summarised in Figure 2.

## 6. Autophagy in Pregnancy Complications

The precise regulation of placental autophagy is crucial to successful pregnancy. When this pathway is dysregulated, autophagy no longer supports healthy placental development but instead drives placental dysfunction and severe pregnancy complications, including PE, FGR, GDM, PTB and RSA. The dysregulated autophagic characteristics and key molecular markers of these pregnancy complications are summarised in Table 2.

### 6.1. Pre-Eclampsia (PE) & Foetal Growth Restriction (FGR)

PE, characterised clinically by new-onset gestational hypertension and proteinuria, is generally linked to uteroplacental malperfusion caused by trophoblast dysfunction, including EVT invasion failure and defective spiral artery remodelling, which then results in oxidative stress and hypoxia at the maternal–foetal interface [9,49]. In this context, autophagy dysregulation is a central pathological feature, though research presents contradictory findings regarding its exact nature [9,41,48], suggesting its role is highly context- and stage-dependent. Several studies reported heightened autophagic markers, including increased LC3 puncta alongside p53 expression, potentially reflecting impaired autophagic clearance rather than enhanced flux, in early-onset PE with or without coexisting FGR [30,41,48]. Under these conditions, pathological overactivation of autophagy restricts trophoblast invasion and leads to shallow placental implantation. In contrast, other work demonstrated reduced autophagy flux and impaired lysosomal biogenesis in term PE placentas; similarly, animal models with trophoblast-specific autophagy deficiency exhibit p62 accumulation and the development of gestational hypertension [11]. Consistent with the study, oxidative stress induced advanced oxidation protein products (AOPPs), suppressed autophagy flux in trophoblasts via the p53/mTOR/p70S6K pathway and induced a senescent phenotype that accelerates placenta ageing and functional decline, which eventually leads to PE [63]. The apparent contradictions in the PE literature may be reconciled by considering disease subtype, early- vs. late-onset PE; placental region, basal plate vs. villous; and methodology, static markers vs. dynamic flux measurement. We propose that PE is characterised not by a uniform defect but by autophagic imbalance, representing a disturbance of the finely regulated homeostatic set point, rather than a straightforward increase or decrease in autophagic activity. Also, it is important to note that while PE and FGR frequently coexist, they may display distinct autophagic signatures. In PE without FGR, autophagic impairment may primarily affect vascular remodelling [64]; in FGR without PE, the defect may lie more in nutrient transport and metabolic adaptation [68,69]. Future studies should stratify these conditions separately. Collectively, these data suggest that autophagy can be either pathologically overactivated to promote trophoblast death and invasion failure, or it can be functionally impaired at the level of lysosomal clearance, resulting in toxic p62 accumulation and defective angiogenic signalling in PE, depending on the disease subtype and the specific placental tissue compartment examined [11,12,48,63].

### 6.2. Gestational Diabetes Mellitus (GDM)

The maladaptive autophagic responses triggered by hyperglycaemic conditions discussed in the previous section appear to further compound in GDM, where persistent maternal hyperglycaemia and insulin resistance can chronically sustain dysfunctional autophagy and thereby amplify the metabolic stress that contributes to pathological placental outcomes like GDM. GDM is characterised by a complex autophagic signature, driven by the temporary development of hyperglycaemia resulting from insulin resistance and maternal pancreatic β-cell dysfunction that occurs during pregnancy [70,71]. HG conditions, a defining metabolic feature of GDM, are known to induce a complex cascade of mitochondrial and ER stress, altering the survival and function of EVTs. This process is mediated by specific molecular mediators, such as p66Shc and DAPK3, which promote autophagy and apoptosis [53,54]. Research indicates that HG reinforces both the expression and mitochondrial translocation of p66Shc within cells, promoting the formation of MAMs and triggering the initiation of cGAS/STING-mediated autophagy and cellular senescence in response to mitochondrial DNA (mtDNA) stress [53]. In parallel, HG upregulates DAPK3, a kinase that facilitates autophagosome-lysosome fusion by regulating the assembly of the STX17-SNAP29-VAMP8 complex [54]. These combined effects of excessive autophagy and mitochondrial damage ultimately lead to massive apoptosis events in the placenta [52]. By contrast, in GDM pregnancies that result in large-for-gestational-age (LGA) infants, autophagy and apoptosis are decreased instead, suggesting that the placental autophagic programme may shift towards a more proliferative and less catabolic phenotype in those with foetal overgrowth [65]. Taken together, these findings suggest that the autophagic signature in GDM is not uniform but varies based on clinical subtypes and glycaemic control. To reduce these discrepancies, future in vitro studies should standardise glucose concentrations and exposure durations to better mirror the magnitude and chronicity of clinical hyperglycaemia in GDM, rather than relying on acute, single supraphysiological HG doses. A critical caveat is that many in vitro studies use supraphysiological glucose concentrations typically in the range of 25 to 50 mM, which do not reflect the mild to moderate hyperglycaemia seen in GDM, where fasting glucose is generally 5.1 mM or higher. This discrepancy may exaggerate autophagic and apoptotic responses, and the relevance of these findings to clinical GDM remains to be confirmed.

### 6.3. Preterm Birth (PTB) & Recurrent Spontaneous Abortion (RSA)

PTB and RSA, or recurrent miscarriages, are strongly associated with defective autophagic defence mechanisms [42,55]. PTB refers to the birth of infants before the completion of 37 weeks of pregnancy, with the majority of these cases caused by intrauterine and placental infections that trigger severe inflammation [72]. Research has confirmed a negative correlation between PTB and autophagy, specifically highlighting the ATG16L1 gene, which plays a key role in trophoblast antibacterial defences against pathogens [55]. A deficiency in this gene increases the ST susceptibility to infections and can synergise with inflammatory stimuli to trigger premature delivery. Similarly, in RSA, clinically defined as the condition of having two or more recurrent miscarriages before 24 weeks of pregnancy [73], autophagy suppression in trophoblasts increases the cytotoxicity of dNK cells and decreases the expression of PEG10 [42]. Autophagy suppression is therefore closely associated with impaired invasive capacity of EVTs, directly compromising successful placentation and contributing to pregnancy loss.

### 6.4. Obstetric Antiphospholipid Syndrome (OAPS)

Obstetric antiphospholipid syndrome (OAPS), a systemic autoimmune disease mediated by circulating antiphospholipid antibodies (aPLs), specifically pathogenic anti-β_2_ glycoprotein I (aβ_2_GP-I) autoantibodies, is a major cause of PE, FGR and RSA [66,67]. At the maternal–foetal interface, recent evidence reveals that OAPS-associated placental pathology is mediated by a severe dysregulation of autophagic homeostasis. Unlike other complications characterised by autophagic suppression, clinical decidual tissues from OAPS patients exhibit pathologically excessive autophagy in EVTs, evidenced by the increase in LC3B-II and concurrent decrease in p62 expression [66,67]. Similarly, exposure of EVTs to aPL antibodies or serum from OAPS patients directly induces this overactivated autophagic state, suppressing cell viability, proliferation, migration, invasion, and endothelial-like tube formation [66,67]. Mechanistically, aPL-mediated excessive autophagy downregulates pro-invasive MMPs, such as MMP-2 and MMP-9, while upregulating invasion-negative regulators like tissue inhibitor of metalloproteinase 2 (TIMP2), leading to shallow invasion and defective spiral artery remodelling [67]. This local trophoblast injury is further amplified by circulating platelet- and endothelial-derived extracellular vesicles (EVs) in OAPS patients; these EVs transfer tissue factor, annexin V, and pathogenic autoantibodies, promoting vascular thrombosis and local inflammation [62]. Crucially, this pathway offers a potential pharmacological target. Hydroxychloroquine (HCQ), commonly used empirically in OAPS, acts as a direct autophagy inhibitor by blocking autophagosome-lysosomal fusion [66,67]. Notably, administration of HCQ at a clinically relevant concentration of 0.1 µg/mL suppresses this excessive autophagy flux, thereby normalising the MMP2/TIMP2 balance and restoring the EVT invasive capacity [67]. Furthermore, in vivo administration of HCQ in OAPS mouse models normalises placental autophagy markers and significantly reduces foetal resorption rates [66]. Collectively, these findings establish that targeting and inhibiting excessive trophoblast autophagy represents a highly promising, mechanistic therapeutic strategy in OAPS.

### 6.5. Translational Challenges of Animal Model in Placental Autophagy Research

A significant challenge in translating placental autophagy phenotypes is the structural and physiological discrepancies between rodent and human placentation [2,6]. Murine placentas exhibit a hemotrichorial barrier with three layers, consisting of two ST layers and a CT layer underneath. In contrast, human placentas consist of a hemomonochorial barrier with only a single ST layer in direct contact with maternal blood [2,6,74,75]. Furthermore, while human EVTs undergo both interstitial and endovascular invasion into the decidua and inner third of the endometrium, rodent trophoblast giant cells (TGCs) and glycogen trophoblasts undergo relatively shallow decidual infiltration [5].

Consequently, although trophoblast-specific deletions of essential autophagic genes, such as *ATG5* and *ATG7*, in mouse models demonstrate that autophagic deficiencies lead to gestational hypertension, severe FGR and altered uterine spiral artery remodelling, these models cannot fully mimic the spatiotemporal cellular remodelling and deep spiral artery transformation that characterise human placentation [11]. Nonetheless, these rodent models are necessary for identifying mechanistic insights since human tissues are not available due to ethical and practical reasons. Thus, while rodent knockout models are indispensable for proving in vivo functionality, their phenotypes must be translated with caution when evaluating human pathologies such as early-onset PE and GDM.

### 6.6. From Biomarker Prediction to Therapeutic Intervention: A Three-Tiered Translational Framework

To successfully translate placental autophagy research from laboratory models to clinical application, a rigorous framework that distinguishes between biomarker development, preclinical mechanistic targeting, and therapeutic intervention is required. Currently, these domains exist at extensively different stages of clinical readiness. This first tier, biomarker development, serves as the most clinically advanced domain. Non-invasive, early-pregnancy biochemical panels, such as the maternal sFlt-1/PIGF ratio used to predict PE or metabolomic profiling for GDM, successfully facilitate risk stratification and patient triage [76,77]. However, these markers reflect downstream systemic endothelial injury or metabolic imbalance instead of serving as direct, real-time readouts of placental autophagic flux. To capture these direct cellular dynamics, research must rely on the second tier of preclinical mechanistic targeting. While highly detailed pathways, such as the HG-induced p66Shc translocation, have been successfully mapped in vitro, direct pharmacological targeting of these core intracellular hubs during human pregnancy remains strictly preclinical due to significant systemic maternal–foetal toxicity and teratogenicity [53].

The preclinical safety barrier directly underscores the crucial gap between empirical drug repurposing and selective autophagy targeting, which defines the third tier, therapeutic intervention. Several major therapeutics that are currently being utilised or undergoing clinical evaluation in complicated pregnancies, including metformin for GDM, pravastatin for early-onset PE, and HCQ for OAPS, have been shown in preclinical models to actively modulate trophoblast autophagic pathways [66,67,78,79]. However, since these agents were used clinically for their primary metabolic, vasoactive, or immunomodulatory profiles instead of their specific autophagy-regulatory capacities, their clinical outcomes, such as the limited sFlt-1 reduction observed, cannot be assumed to directly reflect their underlying ‘autophagy-modulating’ therapeutic mechanisms. Consequently, this translational gap highlights the crucial need for more targeted and cautious future research.

## 7. Conclusions

Autophagy functions as a vital homeostatic mechanism throughout placental development, providing nutrients, energy, and protection during metabolic stress to maintain pregnancy success in terms of trophoblast survival, invasion, and vascular remodelling. This review establishes autophagy as a precisely regulated network indispensable for trophoblast differentiation and syncytialization through p53 downregulation and ER stress coordination, while its effects on invasion depend on oxygen tension, autophagic flux integrity, and differentiation state, which may be heavily influenced by the evolutionary balance of genomic imprinting. Under physiological stressors, including ER stress, oxidative stress, hypoxia, and metabolic challenges, autophagy serves as a pivotal cellular adaptor. These observations emphasise that placental autophagy should not be viewed as simply beneficial or detrimental based on activation or inhibition alone; rather, distinct autophagic states carry different biological meanings across placental development and disease. The dysregulation of autophagy underlies major pregnancy complications, including PE, FGR, GDM, PTB, RSA, and OAPS, although it is crucial to recognise that their autophagic signatures vary significantly between different clinical subtypes and metabolic status. A fundamental unresolved question is whether these autophagic changes are causal drivers of pathology or secondary consequences of the disease placental environment. Resolving the causality is a prerequisite for therapeutic targeting.

A further critical gap concerns cell-type specificity, as current studies largely rely on bulk trophoblast populations, obscuring how autophagic activity differs among CTs, STs and EVTs. Given the complicated nature of placental autophagy, experimental models to study its autophagic nature must be rigorously designed. This methodological rigour requires prioritising targeted genetic approaches over non-specific pharmacological modulators, along with the systematic classification of experimental findings by species. Equally underexplored are the temporal dynamics of placental autophagy across gestation, which are critical for determining intervention timing. Despite substantial progress, our current understanding of placental autophagy remains constrained by several critical limitations. Experimental evidence is currently dominated by immortalised trophoblast cell lines, which only partially reflect the phenotype, differentiation trajectories, and stress responses of primary trophoblasts in the in vivo placental environment. Furthermore, species-specific differences in placental architecture, trophoblast lineages, and the maternal–foetal interface complicate the extrapolation of findings from murine models to human pregnancy. Murine and human placentas differ substantially in architecture and trophoblast lineage composition, raising concerns about translational validity. Actively addressing these species-specific limitations is therefore essential for successful clinical translation. To overcome these limitations, future research should pivot towards human-based physiologically relevant models. The implementation of trophoblast stem cells, 3D trophoblast organoids and spheroids, co-culture systems integrating decidua or immune cells, microfluidic ‘placenta-on-a-chip’ platforms, and precision-cut placental explants will better recapitulate human physiology than traditional cell lines or murine models.

Beyond the mechanistic gaps, the therapeutic window for autophagy modulation in pregnancy is another practical challenge. Systemic modulators carry non-trivial foetal safety risks, and trophoblast-targeted delivery strategies need to be developed. Moreover, the absence of pregnancy-specific clinical studies using autophagy-modulating agents leaves a major gap between mechanistic insight and translational application. To bridge this translational gap, we propose a three-tiered framework that systematically distinguishes between early biomarker prediction, preclinical mechanistic targeting, and clinical therapeutic intervention. Although recent multi-omics studies have nominated autophagy-related genes as potential biomarkers in pregnancy complications, these candidates are still at the discovery and validation stages in preclinical and animal models. Their safety, optimal dosing, and efficacy remain untested in pregnant patients, and no autophagy-directed therapy for pregnancy complications has yet undergone successful clinical validation. Future work should prospectively validate autophagy-related biomarker panels with carefully controlled, pregnancy-specific trials that evaluate the safety and therapeutic potential of pharmacologic and nutraceutical autophagy modulators. These integrated efforts will be essential to determine whether the precise tuning of autophagy in trophoblasts can causally reshape the course of pregnancy complications and sustainably improve maternal and foetal outcomes.

## Figures and Tables

**Table 2:** Summary of dysregulated autophagic characteristics, associated placental dysfunctions, and key molecular markers across major pregnancy complications. Adapted from respective authors as cited in the table.

Pregnancy Complications	Placental Dysfunction	Dysregulated Autophagic Mechanism	Key Autophagy Markers and Methodological Evidence	Species/Model System	Key References
Pre-eclampsia (PE)	- Impaired EVT invasion - Shallow placental implantation - Defective spiral artery remodelling - Oxidative stress and hypoxia	- Autophagic imbalance (context-dependent): ○ Pathological overactivation ○ Impaired lysosomal clearance (autophagosome-lysosome fusion blockade)	- ↑ LC3-II & ↑ p62 - (Western blot/immunohistochemistry (IHC)) - ↑ p53 (Western blot & IHC) - ↑ AOPPs (ELISA/western blot)	- Human - Mouse	[9,11,30,41,48,63]
Foetal Growth Restriction (FGR)	- Impaired nutrient transport - Impaired metabolic adaptation	- Impaired autophagic flux: ○ Shares features with PE, but the defect predominantly affects the cellular adaptation to metabolic stress and nutrient deprivation	- ↑ LC3-II & ↑ p62 (Western blot) - ↑ p53 (IHC/IF)	- Human	[30,41,48,64]
Gestational Diabetes Mellitus (GDM)	- ER stress - Mitochondrial dysfunction - Altered cellular adaptation	- Hyperglycemia-driven dysregulation: ○ Excessive autophagy initiation and mitophagy ○ Autophagosome-lysosome fusion blockade	- ↑ p66Shc & cGAS/STING (Western blot/MAM fractionation) - ↑ DAPK3 (SNARE co-IP) - ↓ p62 (in LGA infants; Western blot) - ↑ PRKN/PINK1 (Western blot)	- Human	[52,53,54,65]
Preterm Birth (PTB)	- Severe intrauterine and placental infection - Severe inflammation - Impaired EVT invasion	- Impaired Xenophagy (pathological clearance): ○ ATG16L1 deficiency increased ST susceptibility to infections ○ Defective autophagic clearance of intracellular pathogens	- ↓ ATG16L1 (Western blot)	- Human	[55]
Recurrent Spontaneous Abortion (RSA)	- Impaired EVT invasion - Increased dNK cytotoxicity - Breakdown of maternal immune tolerance	- Suppressed autophagic initiation: ○ Inhibition of autophagic pathways	- ↓ PEG10 (Western blot/qPT-PCR) - ↑ IGF-2 (Western blot/qPT-PCR)	- Human	[42]
Obstetrics Antiphospholipid Syndrome (OAPS)	- Shallow EVT invasion - Impaired migration and tube formation - Defective spiral artery remodelling	- Pathological overactivation: ○ Excessive autophagy in EVTs (independent of apoptosis)	- ↑ LC3B-II & ↑ p62 (Western blot) - ↓ MMP2/9 & ↑ TIMP2 (Western blot)	- Human - Mouse	[62,66,67]

## Data Availability

No new data were created or analyzed in this study. Data sharing is not applicable to this article.

## Abbreviations

The following abbreviations are used in this manuscript:

3-MA	3-methyladenine
aPLs	Antiphospholipid antibodies
aβ_2_GP-I	Anti-β_2_ glycoprotein I
AD-MSC	Amnion-derived mesenchymal stem cell
AMPK	AMP-activated protein kinase
AOPPs	Advanced oxidation protein products
ATG	Autophagy-related
BAF	Bafilomycin A1
BECN-1	Beclin-1
CM	Conditioned medium
CTs	Cytotrophoblasts
CV-MSCs	Chorionic villus-derived mesenchymal stem cells
CQ	Chloroquine
DAPK3	Death-associated protein kinase-3
dNK	Decidual natural killer
dpc	days post-conception
ER	Endoplasmic reticulum
EVTs	Extravillous trophoblasts
EVs	Extracellular vesicles
EZH2	Enhancer of zeste 2 polycomb repressive complex 2 subunit
FGR	Foetal growth restriction
Gal-4	Galectin-4
GDM	Gestational diabetes mellitus
HBEGF	Heparin-binding EGF-like growth factor
HCQ	Hydroxychloroquine
hCG	Human chorionic gonadotropin
HG	High glucose
HIF-1α	Hypoxia-inducible factor 1-alpha
IHC	Immunohistochemistry
IF	Immunofluorescence
IGF-2	Insulin-like growth factor 2
IGFBP5	Insulin-like growth factor binding protein 5
IP	Immunoprecipitation
LGA	Large-for-gestational-age
MAM	Mito-associated ER membrane
MMP	Matrix metalloproteinases
mtDNA	Mitochondrial DNA
mTOR	Mechanistic target of rapamycin
NF-κB	Nuclear factor-kappa B
NP	Normal pregnancy
OAPS	Obstetric antiphospholipid syndrome
O_2_	Oxygen
oTCs	Ovine trophoblast cells
ox-LDLs	Oxidised low-density lipoproteins
p66Shc-KD	p66Shc knockdown
PE	Pre-eclampsia
PEG10	Paternally expressed gene 10
PGC-1α	Peroxisome proliferator-activated receptor γ coactivator-1 alpha
PHTs	Primary human trophoblasts
PINK1	PTEN induced kinase 1
PPARγ	Peroxisome proliferator-activated receptor γ
PRKN	Parkin
PS	Primitive syncytium
PTB	Preterm birth
ROS	Reactive oxygen species
RSA	Recurrent spontaneous abortion
shRNA	Short hairpin RNA
siATG5	Silencing ATG5
siDAPK3	Silencing DAPK3
siIGFBP5	Silencing IGFBP5
siRNA	Silencing RNA
STs	Syncytiotrophoblasts
TE	Trophectoderm
TEM	Transmission electron microscopy
TGCs	Trophoblast giant cells
TIMP2	Tissue inhibitor of metalloproteinase 2
TSCs	Trophoblast stem cells
TUDCA	Tauroursodeoxycholic acid
ULK1	Unc-51 like autophagy activating kinase-1
UPR	Unfolded protein response
vCTs	Villous CTs
β-gal	β-galactosidase
